# Decline in Partner-Accompanied Births during the COVID-19 Pandemic in Japan: A Nationwide Cross-Sectional Internet-Based Study

**DOI:** 10.3390/ijerph20054546

**Published:** 2023-03-03

**Authors:** Mai Uchida, Sumiyo Okawa, Yoshihiko Hosokawa, Takahiro Tabuchi

**Affiliations:** 1 Department of Psychiatry, Massachusetts General Hospital, Boston, MA 02114, USA; 2 Department of Psychiatry, Harvard Medical School, Boston, MA 02115, USA; 3 Institute for Global Health Policy Research, Bureau of International Health Cooperation, National Center for Global Health and Medicine, Tokyo 162-8655, Japan; 4 Department of Obstetrics and Gynecology, Faculty of Medicine, University of Tsukuba, Tsukuba 305-8577, Japan; 5 Cancer Control Center, Osaka International Cancer Institute, Osaka 541-8567, Japan

**Keywords:** birth partner, parenting, mental health, COVID-19

## Abstract

The study objective was to describe trends in partner-accompanied birth between January 2019 and August 2021 and examine the associations of partner-accompanied birth with women’s psychological distress and partners’ housework and parenting. A total of 5605 women who had a live singleton birth between January 2019 and August 2021 and had a partner participated in this nationwide internet-based survey between July and August 2021 in Japan. The percentages of women’s intentions and actual experience of partner-accompanied births were calculated per month. Associations of partner-accompanied birth with scores on the Kessler Psychological Distress Scale (K6) ≥10, partners’ participation in housework and parenting, and factors associated with having a partner-accompanied birth were examined using a multivariable Poisson regression model. The proportion of women who had partner-accompanied births was 65.7% between January 2019 and March 2020, dropping to 32.1% between April 2020 and August 2021. Partner-accompanied birth was not associated with a K6 score ≥10, but was significantly associated with the partner’s daily housework and parenting (adjusted prevalence ratio 1.08, 95% CI 1.02–1.14). Partner-accompanied births have been substantially restricted since the beginning of the COVID-19 pandemic. The right to a birth partner should be protected, while addressing infection control.

## 1. Introduction

Since the declaration of the COVID-19 pandemic in early 2020, medical institutions worldwide have grappled with ways to provide the necessary medical care while protecting their patients and staff from the infectious disease. One difficult decision that hospitals have faced is whether to allow the accompaniment of birth partners (i.e., partners, doulas) during labor and delivery.

The World Health Organization (WHO) has issued a strong recommendation supporting women’s right to have a chosen companion during labor and delivery, even during the COVID-19 pandemic [1]. Studies have shown that a woman’s access to trusted emotional, psychological, and practical support has been associated with reductions in emergency cesarean births, instrumental vaginal births, and the need for oxytocin augmentation [2,3,4,5,6]. Birth partners’ support during labor and delivery has also been associated with Apgar scores ≥8, a widely used method for reporting the health status of newborns immediately after birth [7] and overall satisfaction with the birth [5]. Based on these factors, in May 2020 the WHO recommended that women be allowed to be accompanied by their partner during labor upon testing the birth partners for COVID-19 and educating mothers and birth partners on appropriate use of personal protective equipment and movement restriction in the facilities [8].

Despite the WHO’s strong recommendations, many countries have reported inconsistencies regarding the restrictions on birth partners. In a survey, around 33% of US women reported that they were not permitted to have a birth partner due to the pandemic [9]. In a study from the UK, 62% of surveyed mothers reported that they were allowed a birth partner during delivery, 18% were unsure of the regulations, and 4% were not permitted accompaniment [10]. These inconsistencies elucidate the difficulties that institutions face in making decisions in the context of risking infection, and these institutional decisions could be incompatible with patients’ desires. The issue calls for further investigation to understand the situation surrounding how accompaniment of birth partners has been handled internationally during the COVID-19 pandemic. Examining the reality of partner-accompanied births and understanding the factors that prevented or promoted partner-accompanied births could aid future planning of the labor and delivery wards to best support laboring mothers.

Our study aimed to examine the level of access to birth partners for mothers who experienced labor and delivery during the pandemic in Japan. Additionally, the study aimed to identify obstetric and family function-related factors associated with the presence or absence of a birth partner. Given the importance of emotional and practical support for mothers during delivery, understanding the impact of COVID-19 on mothers’ access to birth partners has clinical and public health implications.

## 2. Materials and Methods

### 2.1. Study Design and Participants

**Figure 1:**
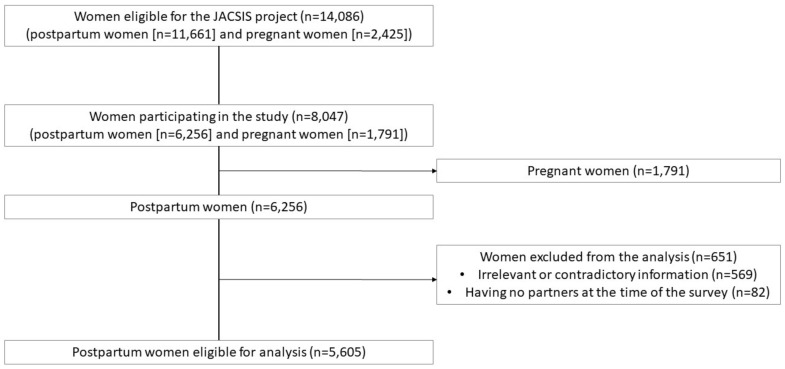
Flowchart of recruitment of the study sample.

This was a nationwide cross-sectional internet-based survey conducted as part of the Japan COVID-19 and Society Internet Survey (JACSIS). The study participants were sampled from the pooled panels of an internet research agency (Rakuten Insight, Inc.), which had approximately 2.2 million panelists registered as of 2019 [11]. This study targeted postpartum women who had a live singleton birth between January 2019 and August 2021 and had a partner, regardless of their marital status. The minimum sample size required was 345 for women who had partner-accompanied births between January 2019 and March 2020 and 335 women who had partner-accompanied births between April 2020 and August 2021. The calculation was performed based on the population of 1,075,000 and 1,172,000 of women who had live births between January 2019 and March 2020 and between April 2020 and August 2021 in Japan [12,13,14]. Calculation anticipated a frequency of 66% and 32% for women who had partner-accompanied births between January 2019 and March 2020 and for women who had partner-accompanied births between April 2020 and August 2021, respectively. A confidence limit of 5% and design effect of 1.0 was anticipated using the OpenEpi online software [15]. Because the JACSIS project addressed various research topics on pregnant and postpartum women and COVID-19, we sampled the maximum number of eligible women from the pooled panels. First, a screening survey was conducted to identify 14,086 eligible women (11,661 postpartum and 2425 pregnant). Then, the survey invitation was sent to all participants via email. Data were collected between July 28 and August 30, 2021, and 8047 women (6256 postpartum and 1791 pregnant) consented to participate in the questionnaire. Of these postpartum women, 5605 were included in the analysis and 651 were excluded from the analysis (569 provided irrelevant or contradictory information, and 82 had no partners at the time of the survey) (Figure 1). The distribution of the participating women per prefecture of residence at the time of the survey nearly corresponded to that of the number of births in 2020 per prefecture (Supplementary Materials Table S1) [16].

### 2.2. Outcome Measures

#### 2.2.1. Partner-Accompanied Birth

We asked the women about their opinion during pregnancy on a partner-accompanied birth (“Did you wish to have a partner-accompanied birth during pregnancy?”) and their experience of whether they had a partner to accompany them during the birth (“Did your partner accompany you during the birth?”). In this study, partner-accompanied birth was defined as having a “partner in life,” regardless of marital status, accompany the labor and delivery process.

#### 2.2.2. Psychological Distress

Psychological distress was measured using the Japanese version of the Kessler Psychological Distress Scale (K6) [17,18,19]. The K6 comprises six items, and the score for each item ranges from 0 to 4 (0 = none of the time, 1 = a little of the time, 2 = some of the time, 3 = most of the time, and 4 = all of the time). Higher scores indicate more distress, with a maximum score of 24. In this study, the cutoff score was 10, which is considered as suspected psychological distress in the national representative Comprehensive Survey of Living Conditions [20]. The Cronbach alpha for the study sample was 0.981, which indicated the reliability of the scale met the standard [21].

#### 2.2.3. Partner’s Participation in Housework and Parenting

The question regarding partner participation in housework and parenting was “Does your partner contribute to housework and parenting?” The response options were “always,” “sometimes,” “not very much,” and “not at all.” The responses were categorized into “always” or “not always.”

### 2.3. Covariates

The covariates were selected based on previous studies on this topic [9,10,22]. These were date of delivery (January 2019–March 2020, April–December 2020, January 2021–August 2021); age (20–24, 25–29, 30–34, 35–47); educational attainment (high school or lower, college/university/postgraduate); whether a woman is cohabiting with a partner (yes, no); whether the woman is currently working (yes, no); household income by quartile (Q1 [<5 million JPY], Q2 [5–6.6 million JPY], Q3 [6.7–8.4 million JPY], Q4 [≥8.5 million JPY], don’t know or want to answer); the number of live births that the woman had had previously, namely parity of live births (1, ≥2); whether the woman had complications during pregnancy (i.e., worsened preexisting illness, hypertensive disorders of pregnancy, pregnancy proteinuria, gestational diabetes, threatened abortion requiring hospitalization, threatened premature labor requiring hospitalization, placenta previa, early abruption of placenta, premature rupture of membrane, and other complications requiring hospitalization); history of depression before or during pregnancy (yes, no); whether her partner attended parenting classes during the antenatal period (yes, no); whether her partner was working from home at least once a week at the time of survey (yes, no); whether the delivery facility was located in the prefectures where the state of emergency for COVID-19 was declared in both 2020 and 2021 by the national government (yes, no); types of delivery facility (hospital, obstetric clinic, midwifery clinic/other); whether the woman delivered and stayed during the peripartum period in their region of origin where her parents may reside, called *satogaeri shussan* in Japanese (yes, no); and mode of delivery (vaginal delivery, planned cesarean section, emergency cesarean section).

### 2.4. Statistical Analysis

Descriptive analysis was performed to summarize the distribution of study participants’ basic characteristics. The percentage of women who had the intention to give birth accompanied by their partner and who had actually done so was calculated per month (between January 2019 and August 2021) to describe the trend over time. The number of participants who delivered between January and June 2019 and August 2021 was small. Thus, we grouped them as those who delivered between January and July 2019 and between July and August 2021. Further, the associations between partner-accompanied birth and postpartum outcomes (i.e., suspected psychological distress [K6 scores ≥10], partners’ daily housework and parenting) and factors associated with partner-accompanied birth were examined using multivariable Poisson regression models with adjustment for the aforementioned covariates. We used Poisson regression models because the prevalence of the outcomes exceeded 10% [23]. For the analysis of the association between accompanied birth and partners’ daily housework and parenting, the variable of history of depression was excluded, because the variable of suspected psychological distress at the time of the survey was included in the analysis. For the analysis of the factors associated with partner-accompanied birth, the variables of current working status of women, whether the partner was currently working from home, their daily housework and parenting, and women’s suspected psychological distress at the time of the survey were not included to avoid reverse causality. *P* < 0.05 was considered statistically significant. All analysis was performed using Stata version 15.1 (StataCorp LLC; College Station, TX, USA).

### 2.5. Ethical Considerations

This study was approved by the Bioethics Review Committee of Osaka International Cancer Institute, Japan (20084). All procedures followed the ethical guidelines for medical and health research involving human subjects enforced by the Ministry of Health, Labor, and Welfare, Japan. Informed consent was obtained electronically before proceeding to the survey, through which all participants were informed that they could withdraw at any time during the study. Their data were collected anonymously and their confidentiality strictly protected. As an honorarium for study participation, the participants received credit points (“Epoints”) after completing the questionnaire.

## 3. Results

### 3.1. Basic Characteristics of Study Participants

Of the 5605 women included in the analysis, 14.9%, 48.4%, and 36.7% delivered between January 2019 and March 2020, April and December 2020, and January and August 2021, respectively (Table 1). The proportion of women who had K6 scores ≥ 10 was 13.6%, and those who reported that their partners undertook housework and parenting daily was 51.2%.

### 3.2. Trend of Partner-Accompanied Birth between January 2019 and August 2021

**Figure 2:**
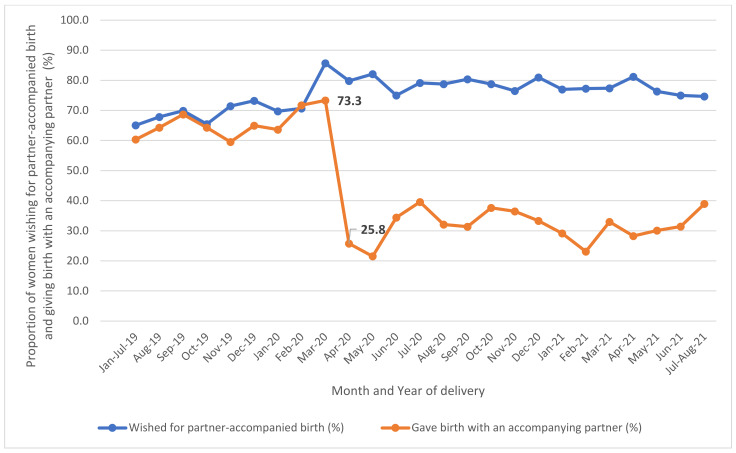
Monthly trend of partner-accompanied birth between January 2019 and August 2021.

The proportion of women who wished for partner-accompanied birth did not change over the observational period, with an average of 71.2% between January 2019 and March 2020 and 78.1% between April 2020 and August 2021 (Figure 2). However, the average proportion of women who gave birth with an accompanying partner was 65.7% between January 2019 and March 2020 and 32.1% between April 2020 and August 2021. A significant gap was observed in April 2020, when the government declared the first state of emergency for COVID-19: 73.3% gave birth with an accompanying partner in March 2020, which dropped to 25.8% in April 2020.

### 3.3. Association of Partner-Accompanied Birth with Suspected Psychological Distress

A K6 score ≥ 10 was observed in 14.2% and 13.2% of women who had their partners accompanying them at birth and those who did not, respectively (Table 2). The adjusted prevalence ratio was not significantly different between the two groups. The ratios were not significant when other cutoff points of the K6 score (i.e., 5 and 13) were used. Among the covariates, partners’ daily housework and parenting were associated with lower reports of suspected psychological distress (aPR 0.49, 95%CI 0.42–0.56). Date of delivery, age of women, current working status of women, household income, and experience of depression before or during pregnancy were also associated with K6 scores ≥10.

### 3.4. Association of Partner-Accompanied Birth with Partner’s Housework and Parenting in the Postpartum Period

Daily housework and parenting by the partner was reported by 52.5% and 50.5% of women who had their accompanying partner at birth and those who did not, respectively (Table 3). The adjusted prevalence ratio of daily housework and parenting by a partner was significantly higher in women who had their accompanying partner during labor and delivery (aPR 1.08, 95%CI 1.02–1.14) than in women who did not. Among the adjustment variables, date of delivery, educational attainment, cohabiting with partner, household income, K6 score, partner’s attending parenting classes during pregnancy, and partner’s working from home were associated with the partner’s undertaking housework.

### 3.5. Factors Associated with Partner-Accompanied Birth

Factors associated with partner-accompanied birth are presented in Table 4. Delivery between April and December 2020 (aPR 0.49, 95%CI 0.46–0.53) and January and August 2021 (aPR 0.45, 95% 0.42–0.49) relative to deliveries between January 2019 and March 2020 showed lower prevalence ratios of partner-accompanied birth consistently, as shown in Figure 1. Maternal factors associated with partner-accompanied birth were maternal age of 35–47 years (aPR 0.81, 95%CI 0.66–0.99) relative to 20–24 years, those with no response to the item on household income (aPR 1.13, 95%CI 1.01–1.26) relative to those in the first quartile of household income, and primipara with live birth (aPR 1.12, 95%CI 1.04–1.20) relative to multipara with live births. Obstetric factors associated with partner-accompanied birth were giving birth at an obstetric clinic (aPR 1.58, 95%CI 1.47–1.69) or midwifery clinic (aPR 1.74, 95%CI 1.48–2.04) relative to a hospital, giving birth and staying in the region where their parents reside (aPR 0.80, 95%CI 0.74–0.86) relative to the regions where the women reside, and planned (aPR 0.48, 95%CI 0.40–0.57) or emergency cesarean section (aPR 0.50, 95%CI 0.42–0.60) relative to vaginal delivery. Giving birth in the facility located in the prefectures where the state of emergency for COVID-19 was declared in 2020 and 2021 was not associated with partner-accompanied birth.

## 4. Discussion

Our study examined mothers’ level of access to a partner-accompanied birth during the pandemic in Japan and identified obstetric and family function-related factors associated with the presence or absence of a birth partner. Our results identified that partner-accompanied birth rates declined to 32.1% after April 2020 and remained similarly low through our data collection period ending August 2021. Partner-accompanied births were significantly associated with (1) partners’ daily participation in housework and parenting, (2) the birth being their first (i.e., primipara), and (3) births at smaller facilities rather than larger hospitals. Further, the rate of partner-accompanied births was negatively associated with mothers giving birth in their region of origin, where their own parents may reside. There was no significant change in the rate of partner-accompanied births in relation to (1) timing during the pandemic or (2) the infection risk within the region (measured by whether the region was federally declared a state of emergency for COVID-19 in 2020 and 2021). Additionally, partners’ daily participation in housework and parenting was associated with fewer reports of maternal psychological distress.

Only 32.1% of laboring mothers were allowed partner-accompanied childbirth during the pandemic in Japan. Previous research has documented that pregnant women greatly benefit from the presence of a support person of their choice during labor and childbirth to provide physical, emotional, and psychological support. Furthermore, there is strong evidence of better maternal and fetal outcomes when birth partners are present. While infection control is extremely important, it is also vital to balance other potential consequences [24], in this case the physical, emotional, and psychological risk to the mothers who had to undergo labor and delivery without their partners and the health risks to their newborn babies.

Regarding the balance of infection control and the consequences of infection prevention measures, COVID-19 positivity rates within the community should be considered. Our results found that partner-accompanied births remained low throughout the assessed time frame (April 2020 to August 2021) and did not change with location or timing. Despite the declining rates of COVID-19 positivity after the pandemic’s start [25] and before the delta variant surge [26], it appears that hospital policies concerning birth partners were not adjusted to reflect lower infectious rates despite regions reporting zero new cases. Additionally, while 21 out of 47 prefectures (i.e., administrative regions) in Japan declared a state of emergency due to high COVID-19 positivity rates in 2020 and 2021 and other prefectures did not, it appears that the community positivity rates were not considered for the allowance of birth partners. Rather, it appears that birth partner accompaniment was uniformly restricted at similar levels, regardless of the community infection risk [27,28]. In contrast, smaller obstetric or midwifery facilities were found to have higher rates of accompanied births. Smaller facilities follow more fluid policies, whereas larger hospitals likely applied strict policies as perinatal medical centers in the community. Considering the benefits of accompanied births, more nuanced and fluid restrictions in response to the infectious risks of the time and regions should be recommended for all medical institutions that manage labor and delivery.

Another factor associated with higher rates of accompanied births was partner involvement in housework and parenting. Our study documented that partner involvement was associated with fewer reports of maternal psychological distress. Japan has ranked 89 out of 189 countries in the Women’s Workplace Equality Index [29], and the Organization for Economic Co-operation and Development (OECD) has reported that women perform unpaid housework and childcare 5.5 times longer than men in Japan, a ratio much higher than that of other OECD countries [30,31], suggesting overall gender inequality at home and in the workplace. Our results suggest that the gender-assigned roles surrounding daily parenting and housework may extend to the presence or absence (of men in heterosexual marriages) in supporting their partners during childbirth. Our results show that only 65.7% of women had partners present at childbirth, even prior to the pandemic, compared to almost universal practice in the US and 90% in the UK [32]. We strongly recommend educating partners in their role of supporting the physical and mental well-being of their partner and baby. We also recommend educating pregnant women about their rights to a birth companion of their choice and encouraging discussions among medical professionals and obstetric facilities to evaluate the benefits of birth partners.

Our results show that pregnant women who returned to their region of origin for childbirth had lower rates of partner-accompanied birth. It is unclear whether these women had other support persons present, such as their families. We hypothesized that when pregnant mothers returned to their region of origin, their partners remained in their regular residence, which could have posed a geographical challenge.

Partner-accompanied birth was not associated with suspected maternal psychological distress, although previous studies have reported that women without partner-accompanied births showed a high prevalence of psychological distress [22,33]. A potential explanation for the inconsistent results is that our study measured the “current” psychological status, which was not always in proximity to their childbirth experience, and therefore may be more affected by “current” situations rather than the experience of delivery without their partner. Nevertheless, postpartum depression is a critical maternal health issue. Women in the youngest age-group (20–24 years) and those living with a low household income showed higher rates of suspected psychological distress in this study, which corresponds to other studies [34,35]. Further, women with lower educational attainment and low household income were less likely to have partners who participated in housework and parenting, which would also affect their psychological well-being. Meanwhile, women were more likely to share housework and parenting with their partner if the partner attended parenting classes during the antenatal period or worked from home. This suggests that parenting classes have a critical role of encouraging partners to participate in housework and parenting in the postpartum period. Working from home, which became common following the COVID-19 pandemic, might have increased the opportunity to share housework and parenting.

To our knowledge, this is the first study to describe the trend of partner-accompanied birth across Japan before and during the COVID-19 pandemic using data from a large sample and to demonstrate that partner-accompanied birth was strongly restricted by the pandemic. However, our study has some limitations. First, we limited our assessment of accompaniment at birth to only partners and did not examine the accompaniment of other support persons, such as parents or friends. Second, our study did not include policy changes regarding partner-accompanied birth at the facility level over the observational period, which could have directly affected the change in partner-accompanied birth rate. However, we adjusted for facility type and mode of delivery in the analysis, and our findings partially explained variations of the partner-accompanied birth policy by facility type or mode of delivery. Third, the data collection survey time frame for some responders was inconsistent, which may have affected memory and experience recall. Fourth, the study findings may have been affected by access to the internet, and participation in the study may have been affected by mental health or domestic problems. Finally, our findings do not explain causal relationships due to the cross-sectional design and should be carefully interpreted.

## 5. Conclusions

Our study documented the significantly and consistently decreased rates of partner-accompanied birth during the COVID-19 pandemic in Japan, regardless of the level of community infection risks. While infection control is extremely important, it is also vital to balance it with potential consequences of the infection control measures themselves. A woman’s access to trusted emotional, psychological, and practical support is associated with reductions in various negative childbirth outcomes, and depriving them of such support yields consequences. The WHO continues to strongly recommend respecting the woman’s right to a chosen companion during labor and delivery, even in the pandemic. Considering the consistently low levels of partner-accompanied births in Japan during the pandemic, it could be advised that the regulation surrounding accompaniment in hospitals be more flexibly managed in response to the infection risks of the community and individual.

## Figures and Tables

**Table 1:** Basic characteristics of participating women (*N* = 5605).

	*N*	%
Total	5605	100
Date of delivery		
January 2019–March 2020	834	14.9
April–December 2020	2712	48.4
January 2021–August 2021	2059	36.7
Age		
20–24	173	3.1
25–29	1458	26
30–34	2284	40.7
35–47	1690	30.2
Educational attainment		
High school or lower	995	17.8
College/university/postgraduate	4610	82.2
Cohabiting with partner		
Yes	5468	97.6
No	137	2.4
Currently working		
Yes	1218	21.7
No	4387	78.3
Household income (by quartile)		
Q1	1062	18.9
Q2	1359	24.2
Q3	1106	19.7
Q4	1257	22.4
don’t know or want to answer	821	14.6
Parity of live birth		
Once	3012	53.7
Twice or more	2593	46.3
Had complications during pregnancy		
Yes	1778	31.7
No	3827	68.3
Depression before or during pregnancy		
Yes	394	7
No	5211	93
K6 score at the time of survey		
<10	4843	86.4
≥10	762	13.6
Partner attended parenting class		
Yes	1146	20.4
No	4459	79.6
Partner currently working from home		
Yes	1020	18.2
No	4585	81.8
Partner practicing housework and parenting		
Always	2872	51.2
Not always	2733	48.8
The state of emergency declared in 2020 and 2021 in the prefecture where delivery facility is located		
Yes	4338	77.4
No	1267	22.6
Type of delivery facility		
Hospital	2696	48.1
Obstetric clinic	2730	48.7
Midwifery clinic/others	179	3.2
Place of delivery		
Region where parents reside	2005	35.8
Region where woman resides	3600	64.2
Mode of delivery		
Vaginal delivery	4520	80.6
Planned cesarean section	601	10.7
Emergency cesarean section	484	8.6

**Table 2:** Adjusted prevalence ratios for the association of partner-accompanied birth with suspected psychological distress (K6 ≥ 10).

	K6 Score				K6 Scores of ≥10	
	<10		≥10			
	*N*	%	*N*	%	aPR *	(95%CI)
Total	4843	86.4	762	13.6		
Delivery accompanied by partner						
Yes	1783	85.8	295	14.2	1.00	
No	3060	86.8	467	13.2	0.99	(0.86–1.15)
Date of delivery						
January 2019–March 2020	681	81.7	153	18.3	1.00	
April–December 2020	2332	86.0	380	14.0	0.85	(0.71–1.02)
January 2021–August 2021	1830	88.9	229	11.1	0.74	(0.60–0.92)
Age						
20–24	133	76.9	40	23.1	1.00	
25–29	1258	86.3	200	13.7	0.71	(0.52–0.96)
30–34	1975	86.5	309	13.5	0.68	(0.51–0.92)
35–47	1477	87.4	213	12.6	0.59	(0.43–0.81)
Educational attainment						
High school or lower	831	83.5	164	16.5	1.00	
College/university/postgraduate	4012	87.0	598	13.0	0.97	(0.83–1.14)
Cohabiting with partner						
Yes	4733	86.6	735	13.4	1.00	
No	110	80.3	27	19.7	1.23	(0.88–1.72)
Current working status						
Yes	1003	82.3	215	17.7	1.00	
No	3840	87.5	547	12.5	0.78	(0.67–0.91)
Household income (by quartile)						
Q1	864	81.4	198	18.6	1.00	
Q2	1168	85.9	191	14.1	0.82	(0.68–0.98)
Q3	974	88.1	132	11.9	0.70	(0.57–0.86)
Q4	1123	89.3	134	10.7	0.63	(0.51–0.78)
don’t know or want to answer	714	87.0	107	13.0	0.75	(0.61–0.93)
Parity of live birth						
Once	2588	85.9	424	14.1	1.00	
Twice or more	2255	87.0	338	13.0	0.96	(0.83–1.11)
Had complications during pregnancy						
Yes	1511	85.0	267	15.0	1.00	
No	3332	87.1	495	12.9	0.93	(0.81–1.07)
Depression before or during pregnancy						
Yes	265	67.3	129	32.7	2.46	(2.10–2.89)
No	4578	87.9	633	12.2	1.00	
Partner attended parenting class						
Yes	977	85.3	169	14.7	1.00	
No	3866	86.7	593	13.3	0.99	(0.83–1.17)
Partner currently working from home						
Yes	886	86.9	134	13.1	1.00	
No	3957	86.3	628	13.7	0.91	(0.77–1.09)
Partner practicing housework and parenting						
Always	2623	91.3	249	8.7	0.49	(0.42–0.56)
Not always	2220	81.2	513	18.8	1.00	
The state of emergency declared in 2020 and 2021 in the prefecture where delivery facility is located						
Yes	3733	86.1	605	13.9	1.00	
No	1110	87.6	157	12.4	0.87	(0.74–1.03)
Type of delivery place						
Hospital	2326	86.3	370	13.7	1.00	
Obstetric clinic	2358	86.4	372	13.6	1.01	(0.88–1.15)
Midwifery clinic/others	159	88.8	20	11.2	0.80	(0.53–1.22)
Place of delivery						
Region where parent reside	1747	87.1	258	12.9	1.00	
Region where woman resides	3096	86.0	504	14.0	1.12	(0.98–1.29)
Mode of delivery						
Vaginal delivery	3905	86.4	615	13.6	1.00	
Planned cesarean section	529	88.0	72	12.0	0.91	(0.73–1.15)
Emergency cesarean section	409	84.5	75	15.5	1.08	(0.86–1.34)

Note * aPR: adjusted prevalence ratio estimated by multivariable Poisson regression model.

**Table 3:** Adjusted prevalence ratios for the association of partner-accompanied birth with partner’s participation in housework and parenting.

	Partner Practices Housework and Parenting				Partner Practices Housework and Parenting	
	Always		Not Always			
	*N*	%	*N*	%	aPR *	(95%CI)
Total	2872	51.2	2733	48.8		
Delivery accompanied by partner						
Yes	1090	52.5	988	47.6	1.08	(1.02–1.14)
No	1782	50.5	1745	49.5	1.00	
Date of delivery						
January 2019–March 2020	352	42.2	482	57.8	1.00	
April–December 2020	1370	50.5	1342	49.5	1.23	(1.12–1.34)
January 2021–August 2021	1150	55.9	909	44.1	1.35	(1.22–1.48)
Age						
20–24	80	46.2	93	53.8	1.00	
25–29	779	53.4	679	46.6	1.06	(0.89–1.25)
30–34	1202	52.6	1082	47.4	1.02	(0.87–1.21)
35–47	811	48.0	879	52.0	0.94	(0.79–1.12)
Educational attainment						
High school or lower	455	45.7	540	54.3	1.00	
College/university/postgraduate	2417	52.4	2193	47.6	1.09	(1.02–1.18)
Cohabiting with partner						
Yes	2838	51.9	2630	48.1	1.00	
No	34	24.8	103	75.2	0.51	(0.38–0.69)
Current working status						
Yes	611	50.2	607	49.8	1.00	
No	2261	51.5	2126	48.5	0.94	(0.88–1.00)
Household income (by quartile)						
Q1	489	46.0	573	54.0	1.00	
Q2	688	50.6	671	49.4	1.05	(0.97–1.14)
Q3	580	52.4	526	47.6	1.06	(0.98–1.16)
Q4	698	55.5	559	44.5	1.10	(1.01–1.19)
don’t know or want to answer	417	50.8	404	49.2	1.06	(0.96–1.16)
Parity of live birth						
Once	1566	52.0	1446	48.0	1.00	
Twice or more	1306	50.4	1287	49.6	1.01	(0.96–1.07)
Had complications during pregnancy						
Yes	882	49.6	896	50.4	1.00	
No	1990	52.0	1837	48.0	1.03	(0.98–1.09)
K6 score at the time of survey						
<10	2623	54.2	2220	45.8	1.00	
≥10	249	32.7	513	67.3	0.62	(0.56–0.69)
Partner attended parenting class						
Yes	637	55.6	509	44.4	1.00	
No	2235	50.1	2224	49.9	0.89	(0.84–0.95)
Partner currently working from home						
Yes	597	58.5	423	41.5	1.00	
No	2275	49.6	2310	50.4	0.88	(0.83–0.93)
The state of emergency declared in 2020 and 2021 in the prefecture where delivery facility is located						
Yes	2232	51.5	2106	48.5	1.00	
No	640	50.5	627	49.5	1.02	(0.96–1.09)
Type of delivery place						
Hospital	1389	51.5	1307	48.5	1.00	
Obstetric clinic	1381	50.6	1349	49.4	0.97	(0.92–1.02)
Midwifery clinic/others	102	57	77	43.0	1.09	(0.96–1.25)
Place of delivery						
Region where parent reside	1001	49.9	1004	50.1	1.00	
Region where woman resides	1871	52.0	1729	48.0	1.04	(0.99–1.10)
Mode of delivery						
Vaginal delivery	2315	51.2	2205	48.8	1.00	
Planned cesarean section	311	51.8	290	48.3	1.04	(0.96–1.13)
Emergency cesarean section	246	50.8	238	49.2	1.01	(0.92–1.11)

Note * aPR: adjusted prevalence ratio estimated by multivariable Poisson regression model.

**Table 4:** Factors associated with partner-accompanied birth.

	Delivered Accompanied by Partner				Delivered Accompanied by Partner	
	Yes		No			
	*N*	%	*N*	%	aPR *	(95%CI)
Total	2078	37.1	3527	62.9		
Date of delivery						
January 2019–March 2020	548	65.7	286	34.3	1.00	
April–December 2020	894	33.0	1818	67.0	0.49	(0.46–0.53)
January 2021–August 2021	636	30.9	1423	69.1	0.45	(0.42–0.49)
Age						
20–24	69	39.9	104	60.1	1.00	
25–29	612	42.0	846	58.0	1.00	(0.82–1.21)
30–34	843	36.9	1441	63.1	0.87	(0.72–1.06)
35–47	554	32.8	1136	67.2	0.81	(0.66–0.99)
Educational attainment						
High school or lower	385	38.7	610	61.3	1.00	
College/university/postgraduate	1693	36.7	2917	63.3	1.02	(0.93–1.11)
Cohabiting with partner						
Yes	2029	37.1	3439	62.9	1.00	
No	49	35.8	88	64.2	0.89	(0.73–1.09)
Household income (by quartile)						
Q1	383	36.1	679	63.9	1.00	
Q2	509	37.5	850	62.5	1.02	(0.92–1.13)
Q3	421	38.1	685	61.9	1.11	(1.00–1.23)
Q4	451	35.9	806	64.1	1.06	(0.95–1.18)
don’t know or want to answer	314	38.2	507	61.8	1.13	(1.01–1.26)
Parity of live birth						
Once	1159	38.5	1853	61.5	1.12	(1.04–1.20)
Twice or more	919	35.4	1674	64.6	1.00	
Had complications during pregnancy						
Yes	598	33.6	1180	66.4	1.00	
No	1480	38.7	2347	61.3	1.06	(0.98–1.14)
Depression before or during pregnancy						
Yes	143	36.3	251	63.7	1.00	(0.88–1.13)
No	1935	37.1	3276	62.9	1.00	
Partner attended parenting class						
Yes	507	44.2	639	55.8	1.00	
No	1571	35.2	2888	64.8	0.93	(0.86–1.01)
The state of emergency declared in 2020 and 2021 in the prefecture where delivery facility is located						
Yes	1647	38.0	2691	62.0	1.00	
No	431	34.0	836	66.0	0.93	(0.86–1.01)
Type of delivery place						
Hospital	737	27.3	1959	72.7	1.00	
Obstetric clinic	1252	45.9	1478	54.1	1.58	(1.47–1.69)
Midwifery clinic/others	89	49.7	90	50.3	1.74	(1.48–2.04)
Place of delivery						
Region where parent reside	678	33.8	1327	66.2	0.80	(0.74–0.86)
Region where woman resides	1400	38.9	2200	61.1	1.00	
Mode of delivery						
Vaginal delivery	1872	41.4	2648	58.6	1.00	
Planned cesarean section	110	18.3	491	81.7	0.48	(0.40–0.57)
Emergency cesarean section	96	19.8	388	80.2	0.50	(0.42–0.60)

Note * aPR: adjusted prevalence ratio estimated by multivariable Poisson regression model.

## Data Availability

The data presented in this study are available on request from the corresponding author. The data are not publicly available for confidentiality purposes.

## Supplementary Materials

The following supporting information can be downloaded at: https://www.mdpi.com/article/10.3390/ijerph20054546/s1, Table S1: Distribution of participating women and the number of births in 2020 per prefecture.
