# Supplementary material for: Decline in Partner-Accompanied Births during the COVID-19 Pandemic in Japan: A Nationwide Cross-Sectional Internet-Based Study
Source: Int J Environ Res Public Health. 2023 Mar 3;20(5):4546. doi: 10.3390/ijerph20054546 (PMC10002132; doi:10.3390/ijerph20054546)
Supplement: Supplementary file 1 [file ijerph-20-04546-s001.zip › ijerph-2228853-supplementary.pdf]

Supplementary materials

Table S1. Distribution of participating women and the number of births in 2020 per prefecture

	Study participants n=5,606		Number of births in 2020* n = 840,804	
	N	%	N	%
Hokkaido	211	3.8	29,523	3.5
Aomori	41	0.7	6,837	0.8
Iwate	22	0.4	6,718	0.8
Miyagi	111	2.0	14,480	1.7
Akita	28	0.5	4,499	0.5
Yamagata	35	0.6	6,217	0.7
Fukushima	58	1.0	11,215	1.3
Ibaraki	88	1.6	17,389	2.1
Tochigi	58	1.0	11,807	1.4
Gunma	58	1.0	11,660	1.4
Saitama	326	5.8	47,327	5.6
Chiba	235	4.2	40,168	4.8
Tokyo	667	11.9	99,661	11.9
Kanagawa	397	7.1	60,865	7.2
Niigata	86	1.5	12,980	1.5
Toyama	55	1.0	6,256	0.7
Ishikawa	49	0.9	7,712	0.9
Fukui	29	0.5	5,313	0.6
Yamanashi	31	0.6	5,184	0.6
Nagano	78	1.4	12,864	1.5
Gifu	108	1.9	12,092	1.4
Shizuoka	156	2.8	22,497	2.7
Aichi	466	8.3	55,613	6.6
Mie	65	1.2	11,141	1.3
Shiga	66	1.2	10,437	1.2
Kyoto	127	2.3	16,440	2
Osaka	515	9.2	61,878	7.4
Hyogo	294	5.3	36,952	4.4
Nara	61	1.1	7,831	0.9
Wakayama	40	0.7	5,732	0.7
Tottori	30	0.5	3,783	0.4

Shimane	30	0.5	4,473	0.5
Okayama	87	1.6	13,521	1.6
Hiroshima	167	3.0	19,606	2.3
Yamaguchi	58	1.0	8,203	1
Tokushima	24	0.4	4,521	0.5
Kagawa	36	0.6	6,179	0.7
Hiroshima	61	1.1	8,102	1
Kochi	19	0.3	4,082	0.5
Fukuoka	245	4.4	38,967	4.6
Saga	32	0.6	6,004	0.7
Nagasaki	39	0.7	9,182	1.1
Kumamoto	52	0.9	13,011	1.5
Oita	43	0.8	7,582	0.9
Miyazaki	34	0.6	7,719	0.9
Kagoshima	57	1.0	11,638	1.4
Okinawa	30	0.5	14,943	1.8

*The number of births in 2020 per prefecture were extracted from government data “Summary of the Annual Vital Statistics Monthly Report, 2020” [16].
